# People with intellectual disabilities and the COVID-19 pandemic

**DOI:** 10.1017/ipm.2020.66

**Published:** 2020-05-27

**Authors:** G. Gulati, E. Fistein, C. P. Dunne, B. D. Kelly, V. E. Murphy

**Affiliations:** Graduate Entry Medical School, University of Limerick, Limerick, Ireland (Email: Gautam.gulati@hse.ie); Department of Public Health and Primary Care, University of Cambridge, Cambridge, UK; Graduate Entry Medical School, University of Limerick, Limerick, Ireland; Department of Psychiatry, Trinity College Dublin, Dublin, Ireland; Department of Psychiatry, University College Cork, Cork, Ireland

The COVID-19 pandemic is acknowledged as the greatest public health challenge for a generation. Not everyone is affected equally, however, and certain sectors of the population present particular cause for concern.

There are over 66 000 people with intellectual disabilities in Ireland (Central Statistics Office, 2016). On 20 March 2018, Ireland deposited an Instrument of Ratification with the United Nations (UN) Office of Legal Affairs, thereby facilitating Ireland’s long-awaited ratification of the UN Convention on the Rights of Persons with Disabilities (CRPD). In doing so, Ireland became the last EU Member State to commit explicitly to meeting the rigorous human rights standards prescribed in this seminal international instrument (United Nations, 2006).

Article 11 of the Convention mandates that States ‘shall take…all necessary measures to ensure the protection and safety of persons with disabilities in situations of risk, including situations of armed conflict, humanitarian emergencies and the occurrence of natural disasters’. The COVID-19 pandemic is a humanitarian emergency during which the State must act to protect the rights of people with intellectual disabilities.

Two areas of health service delivery merit particular attention in this context: ensuring equitable access to life-saving treatments and ensuring that healthcare resources are not diverted inappropriately at a time of enhanced need.

With respect to the first issue – equitable access to care - reports from the United Kingdom regarding DNR (‘Do Not Resuscitate’) orders made in respect of people with intellectual disabilities present particular cause for concern (Thomas, 2020; Ryan, 2020). Some of these orders have reportedly been made in situations where the person and their family have not been consulted. Similar reports have been published in respect of elderly patients in Northern Ireland.

While emergency changes to Irish metal health legislation have focussed on the role of mental health tribunals and safeguards pertinent to people in approved centres, it must be stressed that there are no changes to capacity law or the fundamental principle of autonomy in healthcare decisions (Kelly, 2020a). There remains a presumption of decision-making capacity. Even at a time of unprecedented demand for healthcare resources, it is important to emphasise a capacity-based approach, with an individual’s will and preferences informing ‘best interests’ decisions in healthcare on a careful, considered, case-by-case basis.

Article 12 of the CRPD places an onus on ratifying States to take measures ensuring access for persons with disabilities to the support they may require in exercising their legal capacity. It is ethically unacceptable for people with a disability to be targeted with a view to making advance directives in respect of life-saving measures based solely on those disabilities. It would be similarly unacceptable to use criteria arguably based on value judgements that may disadvantage people with disabilities when triaging people seeking access to intensive care facilities (Walker, 2020; Tuffrey-Wijne, 2020). The focus when making such decisions must be on likelihood of surviving intensive treatment and not based on the presence of a disability.

Regarding the second issue – potential inappropriate diversion of resources – it is noteworthy that while COVID-19 presents predominantly as a respiratory syndrome, there is also psychological distress associated with the pandemic. Clearly, this arises in response to fears about personal and familial infection as well as the sequelae of social distancing and quarantine measures (Kelly, 2020b). In this context, people with disabilities are likely to be particularly vulnerable to isolation and psychological distress.

Reports of staff redeployment from mental health and disability services to other health services in response to the pandemic are therefore of considerable concern (Cullen *et al.* 2020; Ford, 2020). People with intellectual disabilities already experience substantial barriers to accessing medical care and are, therefore, likely to be at increased risk of both the infection itself and challenges in accessing information, testing and treatment. Services for people with intellectual disabilities routinely assist with these tasks and can thus impact on infection rates as well as physical and psychological well-being.

Article 25 of the CRPD requires that member States do not discriminate against people with disabilities in the provision of healthcare. This is particularly important when considering resources during a pandemic. Evolving experience from the UK and Ireland suggests that people in care homes and other residential facilities are particularly vulnerable to outbreaks of COVID-19. Potential under-reporting of cases and related deaths in Irish care homes and among people with intellectual disabilities merit close attention (RTÉ News, 2020). Two years on from Ireland’s ratification of the CRPD, the health service response to the COVID-19 pandemic is likely to be a litmus test of the State’s commitment to its professed core values.
